# Supplementary material for: Modular in vivo assembly of Arabidopsis FCA oligomers into condensates competent for RNA 3’ processing
Source: EMBO J. 2025 Feb 24;44(7):2056–74. doi: 10.1038/s44318-025-00394-4 (PMC11962161; doi:10.1038/s44318-025-00394-4)
Supplement: Supplementary file 1 — Appendix [file 44318_2025_394_MOESM1_ESM.pdf]

Appendix for

Modular *in vivo* assembly of *Arabidopsis* FCA oligomers into condensates competent for RNA 3' processing

Table of Contents

Appendix Figure S1. The co-localization of 3D images is achieved after object identification using the blob finder algorithm……………………..2

Appendix Figure S2. An additional experiment analysing the co-localization of FCA and FLL2……..................3

Appendix Figure S3. Stoichiometry distribution of individual FCA particles in FCAwt and FCArrm genotypes in different experiments…………...4

Appendix Figure S4. Integrated nuclear intensity in FCAwt and FCArrm genotypes from different experiments……………………………………………………………………………….………………...……5

Appendix Figure S5. Quantification of FCA particle stoichiometry and diffusion coefficient..……......................6

Appendix Figure S6. SlimVar at 10 ms/frame provides optimal contrast for detection of FCA-mScarlet-I and FCA-eGFP particles.……………………………………………………………………….………….......….7

Appendix Figure S7. Two additional independent DRIPc–qPCR experiments were performed to analyze the *COOLAIR* R-loop in the indicated genotypes………………………………………………………………….8

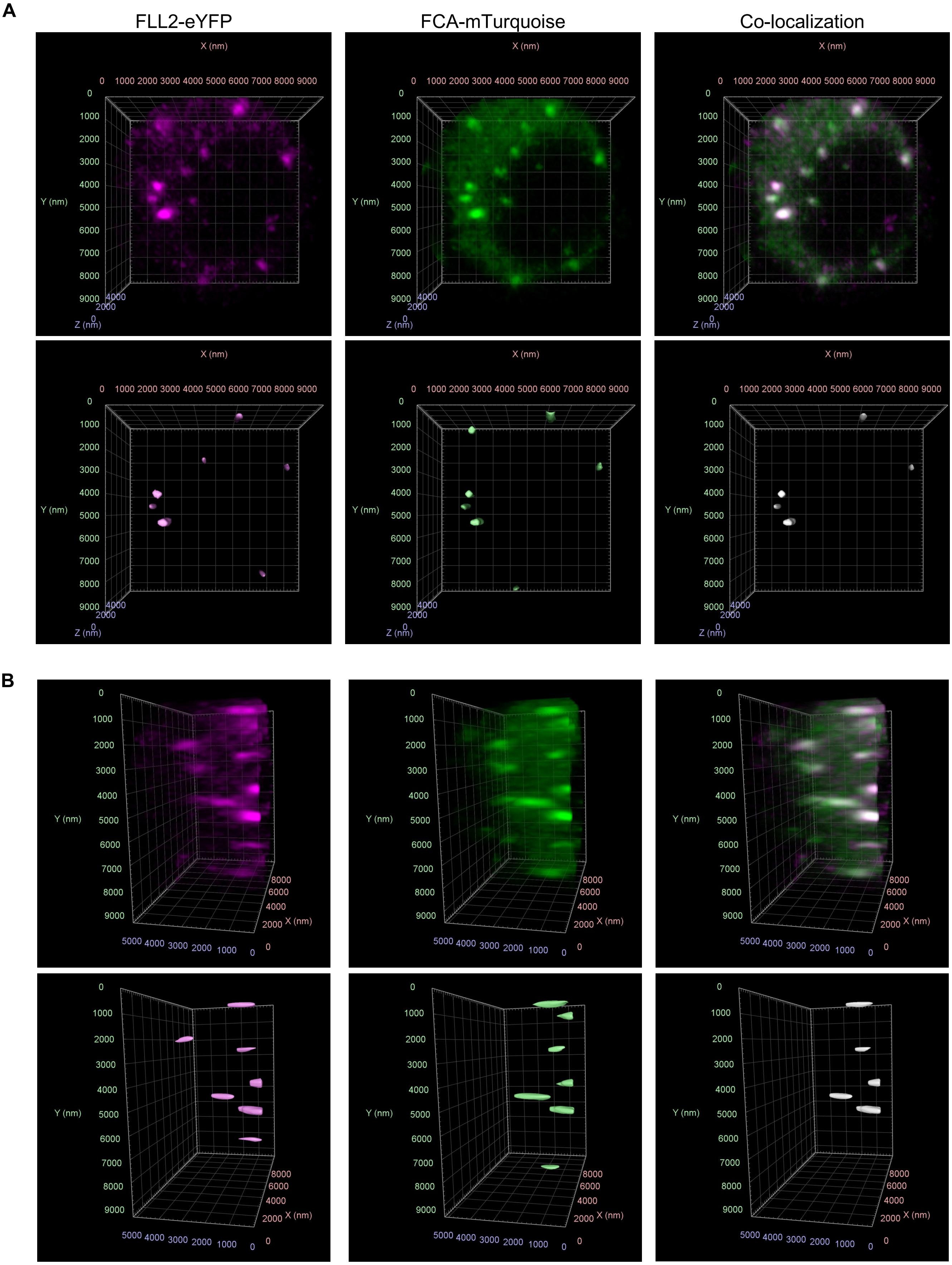

Appendix Figure S1. The co-localization of 3D images is achieved after object identification using the blob finder algorithm

Front view (A) and side view (B) of the 3D image with grid lines, consistent with Figure 2A, illustrating the FCA-mTurquoise, FLL2-eYFP, and the objects identified using the blob finder algorithm, as described in the Methods. In the identified objects, green dots denote FCA-mTurquoise identified foci, magenta dots indicate FLL2-eYFP foci identified, and the white region highlights the co-localized area.

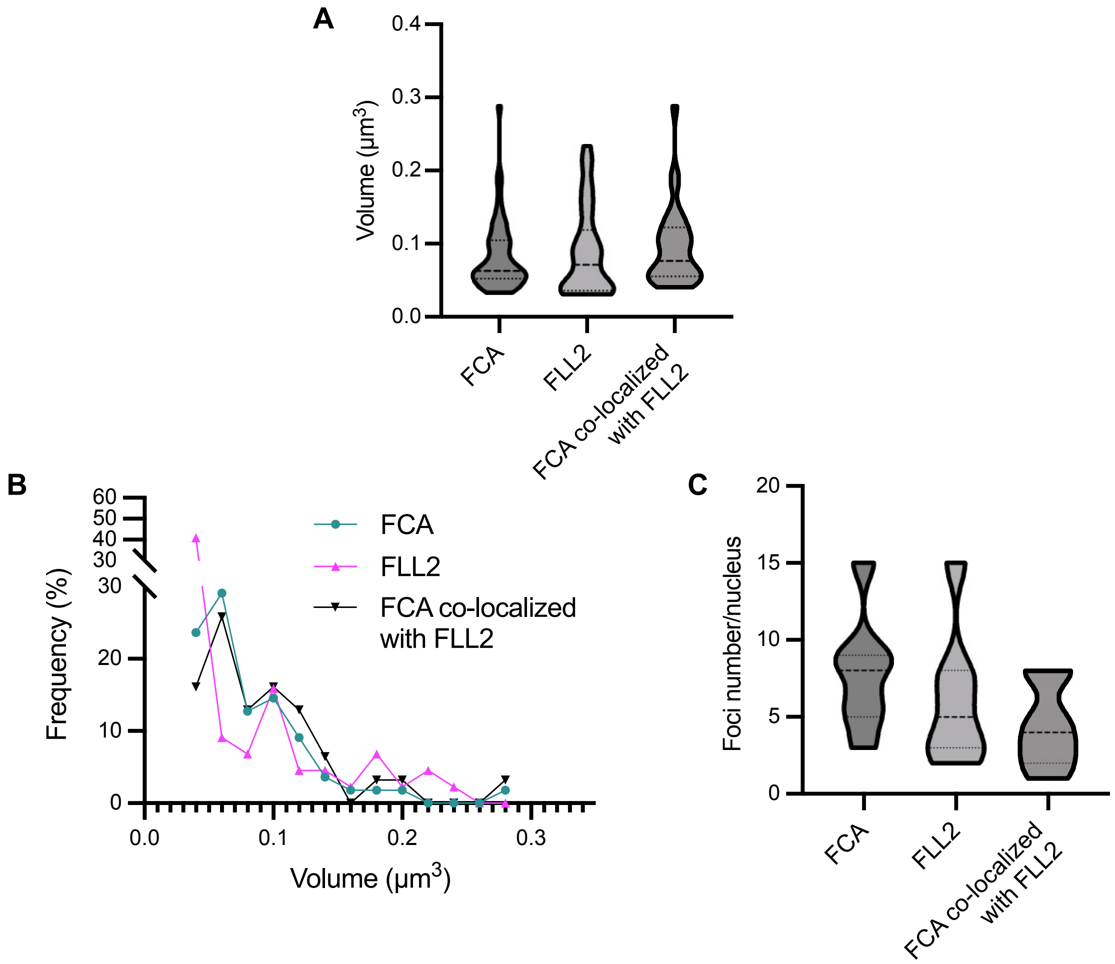

Appendix Figure S2. An additional experiment analysing the co-localization of FCA and FLL2
(A) Quantification of size of FCA (total FCA-mTurquoise identified foci), FLL2 (total FLL2-eYFP identified foci) and FCA identified foci that are co-localized with FLL2. The lines in the violin plots indicate the median and quartiles. (B) Size and frequency distribution of FCA, FLL2 foci and FCA identified foci that are co-localized with FLL2. (C) Number of FCA, FLL2 foci and FCA foci that are co-localized with FLL2 in each nucleus. The lines in the violin plots indicate the median and quartiles. The images for the quantification were acquired on a Zeiss LSM980 using an LD C-Apochromat 63x/NA 1.2 water-immersion objective. The fluorescent proteins, mTurquoise and eYFP, were excited at 458 and 514 nm, respectively. The voxel size of images for quantification analysis was 0.05 μm x 0.05 μm x 0.21 μm (xyz). The quantitative analysis was based on 7 nuclei.

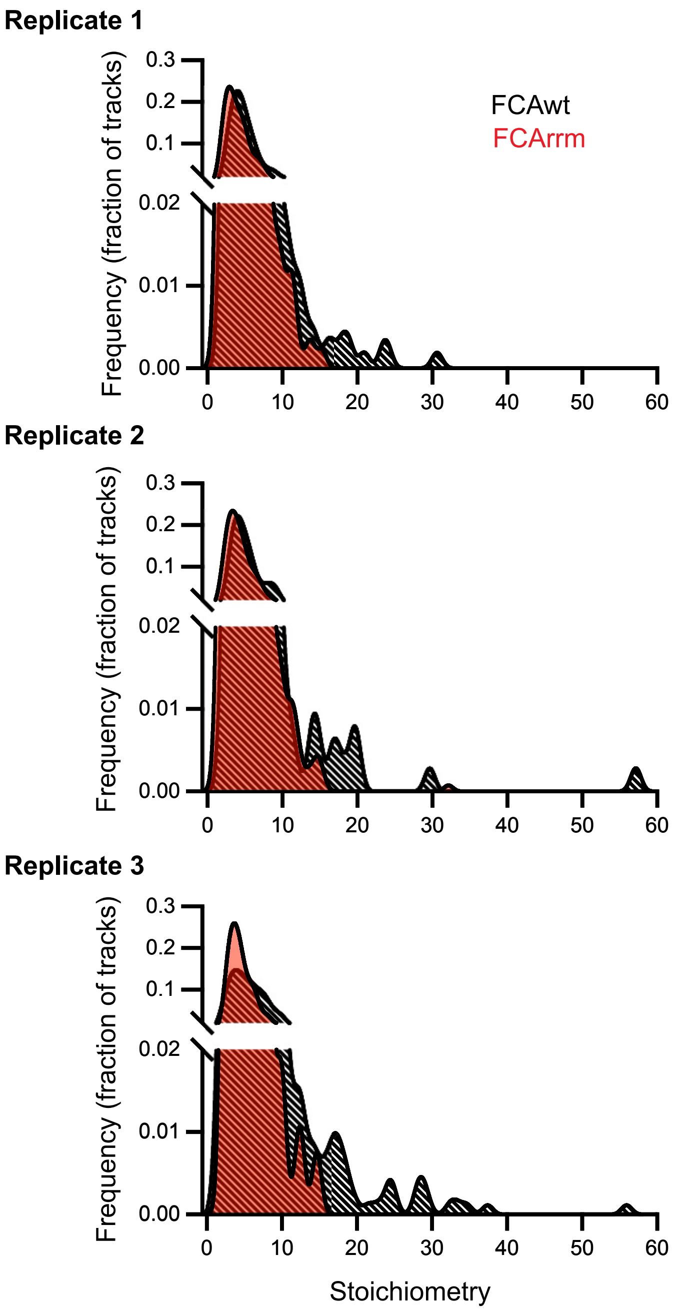

Appendix Figure S3. Stoichiometry distribution of individual FCA particles in FCAwt and FCArrm genotypes in different experiments

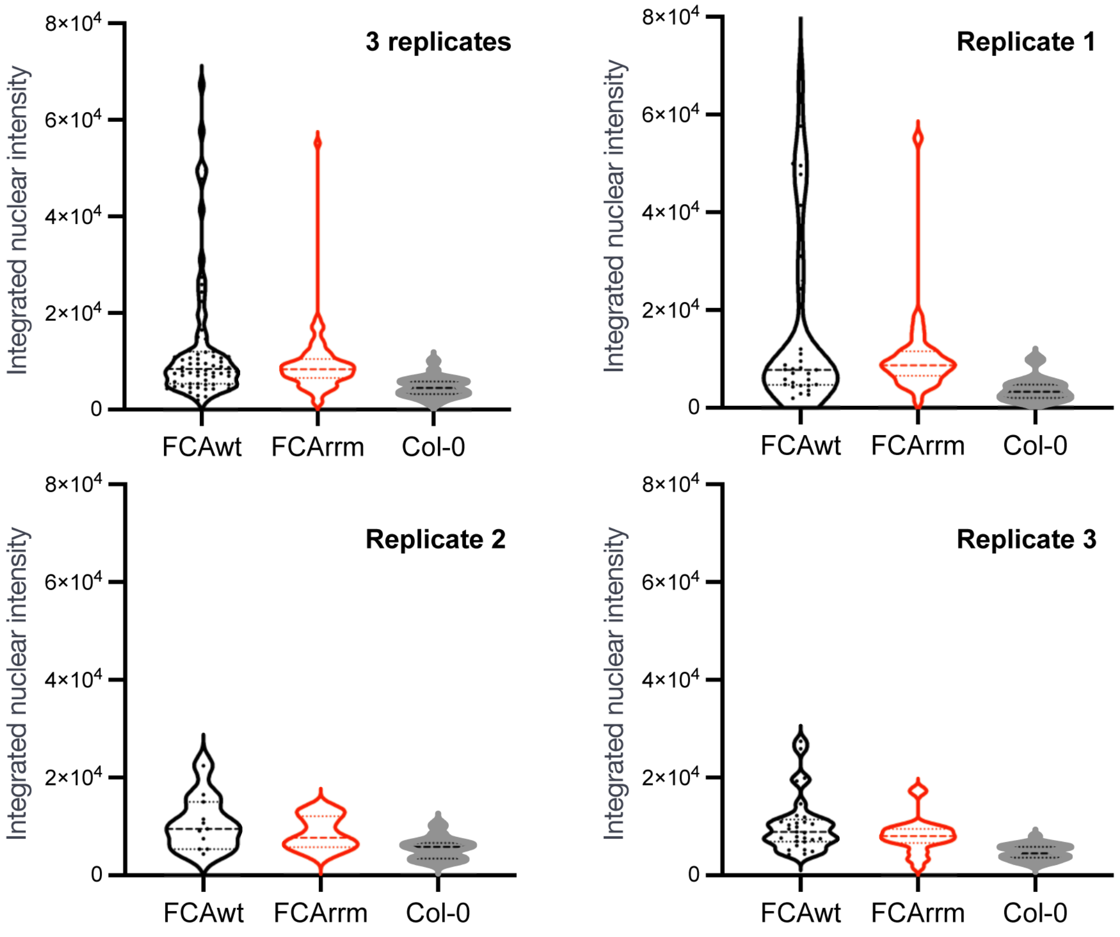

Appendix Figure S4. Integrated nuclear intensity in FCAwt and FCArrm genotypes from different experiments

The violin plots represent the integrated nuclear intensity (in molecules) of FCA reporters in FCAwt and FCArrm from each biological replicate. Col-0 is negative control.

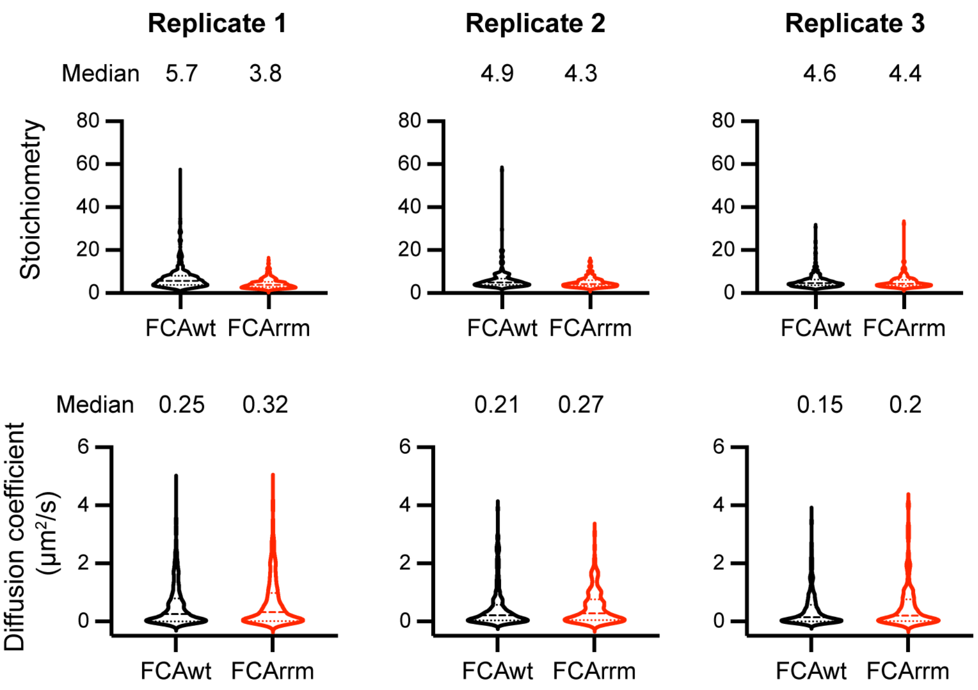

Tracking number				
	Replicate 1	Replicate 2	Replicate 3	Total
FCAwt	504	202	302	1008
FCArrm	560	171	182	913

Appendix Figure S5. Quantification of FCA particle stoichiometry and diffusion coefficient
The violin plots represent the FCA reporter stoichiometry and diffusion coefficient in FCAwt and FCArrm from each biological replicate. The table displays the sample statistics used for quantitative SlimVar analysis, including the numbers of tracks.

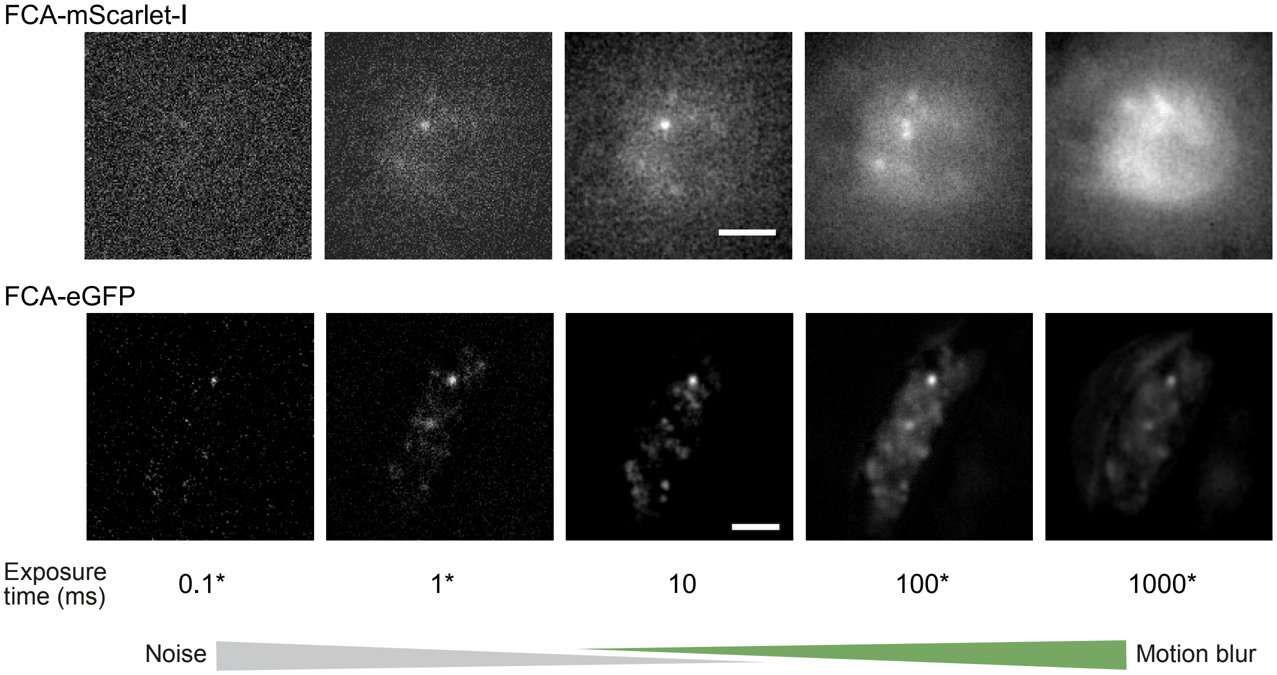

Appendix Figure S6. SlimVar at 10 ms/frame provides optimal contrast for detection of FCA-mScarlet-I and FCA-eGFP particles
A single acquisition at 10 ms/frame exposure was used to simulate a range of exposure times (labelled with *). Longer exposure times were generated by summing 10 or 100 frames directly, while shorter exposures were created by dividing a frame‘s pixel intensity by 10. This was done either with FCA-mScarlet-I or FCA-eGFP. Frames are displayed with individual min/max contrast stretching. Below 1 ms/frame, shot noise reduces the contrast to a level insufficient for observing single molecules, whereas at 100 ms/frame, motion blur and photobleaching degrade the contrast for all but the brightest and least mobile assemblies. Scale bar: 2 µm.

Replicate 2

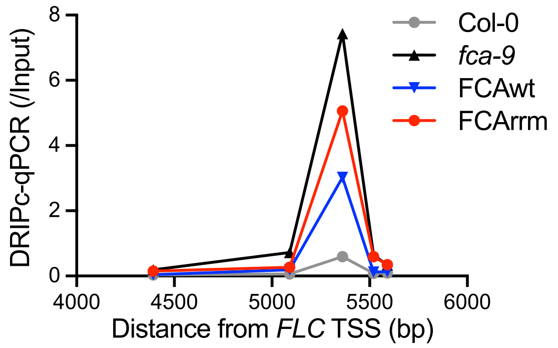

Replicate 3

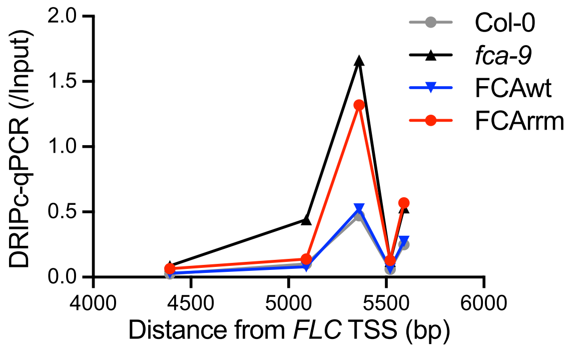

Appendix Figure S7. Two additional independent DRIPc–qPCR experiments were performed to analyze the *COOLAIR* R-loop in the indicated genotypes

Data are presented as mean from four technical repeats for Replicate 2 and three technical repeats for Replicate 3. TSS, transcription start site. The primer sets used for DRIPc-qPCR (A to E) corresponds to that shown in Figure 4D.
