# Supplementary material for: Modular in vivo assembly of Arabidopsis FCA oligomers into condensates competent for RNA 3’ processing
Source: EMBO J. 2025 Feb 24;44(7):2056–74. doi: 10.1038/s44318-025-00394-4 (PMC11962161; doi:10.1038/s44318-025-00394-4)
Supplement: Supplementary file 2 — Table EV1 [file 44318_2025_394_MOESM2_ESM.docx]

Table EV1: Primers

| **Primer name** | **Primer sequence 5' -> 3'** | **Annotations** |
| --- | --- | --- |
| **Primers for cloning** |  |  |
| Inf-XhoI-pFCA-F | CGGGCCCCCCCTCGACGAGCGGTGAAGATGTATTGT | Cloning of FCA genomic fragments with Infusion strategy |
| Inf-BamHI-FCA-3UTR-R | TAGAACTAGTGGATCACAACCTTGAGTTCCTCGAGT |  |
| Inf-FCA-stop-linear-R | AGCTTTATTCTTCCACATGAGTTCTTGG |  |
| Inf-FCA-stop-linear-F | TGAGGTTCATATCTACCCTTTCTCTCCT |  |
| Inf-FCA-linker-mScarletI-F | TGGAAGAATAAAGCTTCTGCTGCCGCATCCGCGGCAGCTTCAGCCGTGAGCAAGGGCGAGGCA | Cloning of FCA-mScarlet-I with Infusion strategy |
| Inf-FCA-mScarletI-R | TAGATATGAACCTCACTTGTACAGCTCGTCCATGC |  |
| Inf-FCA-stop-linear-R | AGCTTTATTCTTCCACATGAGTTCTTGG | Cloning of FCA fusion protein with Infusion strategy |
| Inf-FCA-stop-linear-F | TGAGGTTCATATCTACCCTTTCTCTCCT |  |
| Inf-NdeI-FCA-In3-F | TAGGTGGTGCAAACATATGGA | Cloning of L304F mutation on FCA fragments with Infusion strategy |
| Inf-FCA-L304F-R | AAAAAACTTAAACTCTAGGGTGC |  |
| Inf-FCA-L304F-F | CCCTAGAGTTTAAGTTTTTTGTTGG |  |
| Inf-AfeI-FCA-In6-R | GTAGAATGTCCAAGCGCTT |  |
| **Primers for transcript analyses** |  |  |
| FCAγ-qPCR-F | TCAGTCGTGCAACTGCTCCTC | Spliced *FCAγ* qPCR |
| FCAγ-qPCR-R | GATCCAGCCCACTGTTGTTT |  |
| FLC spliced F | AGCCAAGAAGACCGAACTCA | Spliced *FLC* qPCR |
| FLC spliced R | TTTGTCCAGCAGGTGACATC |  |
| Set1 new LP | TGGTTGTTATTTGGTGGTGTG | RT for proximal *COOLAIR* |
| set2_new_2_LP | CCTGCTGGACAAATCTCCGA | Proximal *COOLAIR* qPCR |
| set2_new_2_RP | TCACACGAATAAGGTGGCTAATTAAG |  |
| FLC -158_F | GCCCGACGAAGAAAAAGTAG | RT for distal *COOLAIR* |
| Set4-new-F-195 | GTATCTCCGGCGACTTGAAC | Distal *COOLAIR* qPCR |
| Set4-new-R-195 | GGATGCGTCACAGAGAACAG |  |
| UBC_qPCR_F | CTGCGACTCAGGGAATCTTCTAA | *UBC* qPCR |
| UBC_qPCR_R | TTGTGCCATTGAATTGAACCC |  |
| **Primers for DRIPc-qPCR** |  |  |
| 4319f | AGAACAACCGTGCTGCTTTT | A |
| 4466r | TGTGTGCAAGCTCGTTAAGC |  |
| 5030F | CCGGTTGTTGGACATAACTAGG | B |
| 5153R | CCAAACCCAGACTTAACCAGAC |  |
| 5327f | TTTTTGTTATGGTTAGGTTTGGA | C |
| 5395r | AGTAGCACTACTTCTAGACACTTGGA |  |
| 5456f | GCTTCCAAACTTAAAAGCTTAAACA | D |
| 5584r | TCTTTTTGTCTTCTATCCAAGGAAT |  |
| 5531F | TGGTTGTTATTTGGTGGTGTG | E |
| 5652R | CTGCTCCCACATGATGATTA |  |
| **Primers for EMSA** |  |  |
| FCA-EMSA-F1-718-T7 | AAGTAATACGACTCACTATAGGGATTTCAACCGCCGATTTAAG |  |
| FCA-EMSA-R-T3 | AAGAATTAACCCTCACTAAAGGGTTCCGGTTGTTGGACATAACT |  |
