# Supplementary material for: Modular in vivo assembly of Arabidopsis FCA oligomers into condensates competent for RNA 3’ processing
Source: EMBO J. 2025 Feb 24;44(7):2056–74. doi: 10.1038/s44318-025-00394-4 (PMC11962161; doi:10.1038/s44318-025-00394-4)
Supplement: Supplementary file 4 — Movie EV1 [file 44318_2025_394_MOESM4_ESM.zip › Movie EV1/Movie EV1 legend.docx]

**Movie EV1.**

A movie corresponding to Fig. 1A.
