# Supplementary material for: Modular in vivo assembly of Arabidopsis FCA oligomers into condensates competent for RNA 3’ processing
Source: EMBO J. 2025 Feb 24;44(7):2056–74. doi: 10.1038/s44318-025-00394-4 (PMC11962161; doi:10.1038/s44318-025-00394-4)
Supplement: Supplementary file 10 — Expanded View Figures [file 44318_2025_394_MOESM10_ESM.pdf]

Expanded View Figures

Figure EV1. A missense mutation (L304F) on the second RRM of FCA attenuates RNA-binding.

(**A**) Schematic representation of the transgenic FCA-mScarlet-I fusion within the FCA locus—the position of the missense L304F mutation is indicated. Untranslated regions are indicated by grey boxes, exon regions by black boxes, and mScarlet-I coding sequence by the red box. (**B**) Expression of *FLC* and *FCA* relative to *UBC* in plants at the T3 generation following transformation. Data represent mean values from technical repeats. The FCAwt #8 and FCArrm #1 lines are used for further analysis. (**C**) FCA protein structure predicted by Alphafold Protein Structure Database (AF-Q5I5A2-F1-v4, Jumper et al, 2021; Varadi et al, 2022). The arrow indicates the location of the amino acid change from the missense mutation (amino acid 213 in this protein structure database corresponds to amino acid 314 when FCA uses the non-canonical start CTG codon). (**D**) Peptide alignment of Arabidopsis FCA proteins from different plant species (https://phytozome-next.jgi.doe.gov). The arrow indicates the conserved Leucine 304. (**E**) Plants carrying transgenes with the L304F mutation show delayed flowering compared to the control. Data are mean ± s.d. ($n = 23$ and 24 seedlings for the control and L304F, respectively). Statistical significance was calculated using the two-tailed t-test, with P-value = 4.4e−23. ****P-value < 0.0001. (**F**) In vitro RNA binding assay showing that the Leu 304 and each RRM domain are important.

 © The Author(s)

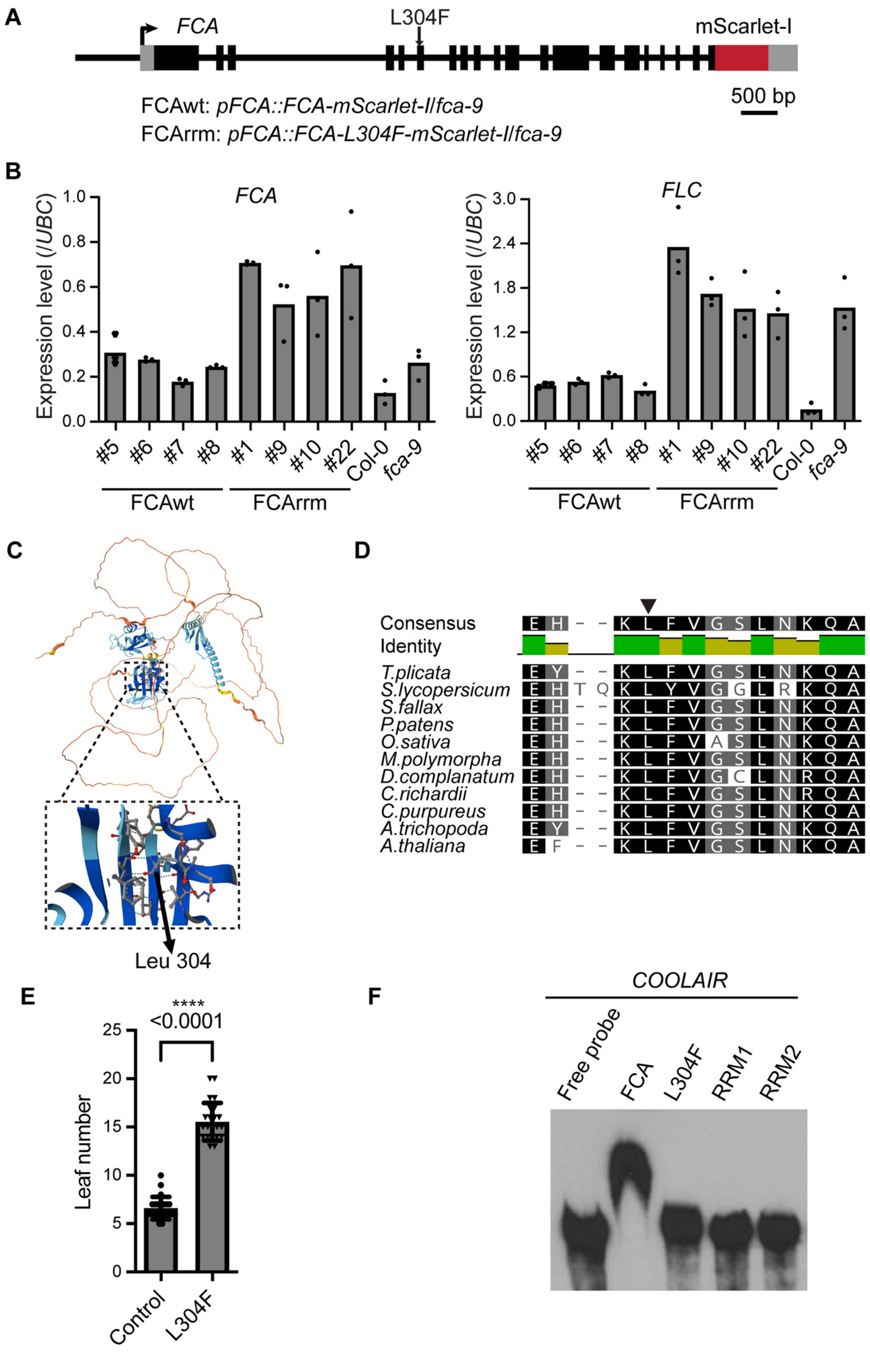
A
FCA
L304F
mScarlet-I
500 bp
FCAwt: pFCA::FCA-mScarlet-I/fca-9
FCArrm: pFCA::FCA-L304F-mScarlet-I/fca-9
B
FCA
Expression level (/UBC)
#5 #6 #7 #8 #1 #9 #10 #22 Col-0 fca-9
FCAwt
FCArrm
FLC
C
Leu 304
D
Consensus
Identity
T.plicata
S.lycopersicum
S.fallax
P.patens
O.sativa
M.polymorpha
D.complanatum
C.richardii
C.purpureus
A.trichopoda
A.thaliana
E
****
<0.0001
Leaf number
Control
L304F
F
COOLAIR
Free probe
FCA
L304F
RRM1
RRM2

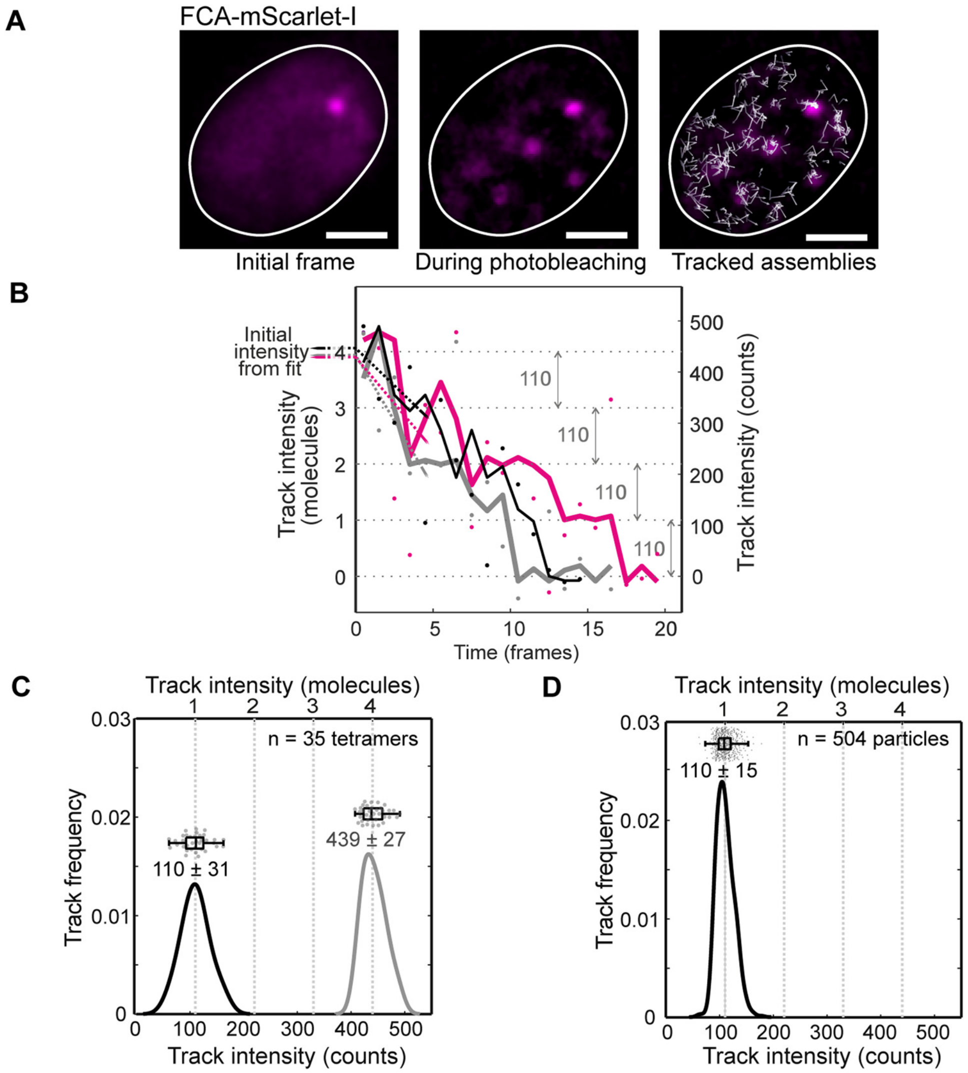

Figure EV2. Automated tracking analysis of FCA particles and determination of the characteristic single-molecule brightness in living tissues.

(**A**) An example of FCA-mScarlet-I particle tracking at 10 ms/frame. The intermediate frame, overlaid with tracks (white arrows) corresponding to the particle paths, achieves super-resolved localization precision and provides estimates of the corresponding stoichiometry and mobility. The left panel shows the initial frame of the acquisition, while the middle panels display the intermediate stage of the acquisition (after 75 ms), where contrast is greatest and assemblies are clearly visible. The nuclear boundary (white closed line) is obtained by blurring the initial frame and setting the Otsu intensity threshold. The third panel features the same detected assemblies throughout the acquisition. Scale bar: 2 μm. (**B**) The consistent photobleaching steps yield an estimate of about 110 photoelectron counts for the characteristic single-molecule brightness of the FCA-mScarlet-I in planta. The intensity of particles near the end of five particle tracks in the FCA-mScarlet-I image sequences is represented by square dots, and the denoised signals by solid lines of the corresponding colour. Here, zero intensity corresponds to the mean background level after complete photobleaching. (**C**) A subset of $n = 35$ particles was identified as having four photobleaching steps. This subset gives a characteristic single-molecule brightness (black line) of 110 ± 31 counts (mean ± s.d., $n = 35$). The initial intensities of these particles (grey line) are each greater than the characteristic single-molecule brightness by a factor of ~ four (stoichiometry: 4.0 ± 0.6 molecules, mean ± s.d., $n = 35$), suggesting that each particle is composed of ~4 FCA-mScarlet-I molecules. Data for individual particles are shown above the corresponding distribution in a jitter and box plot (showing median, interquartile range and min-max range). (**D**) A characteristic single-molecule brightness of 110 ± 15 counts (mean ± s.d., $n = 504$) is calculated from the time-averaged intensity levels of each of $n = 504$ particles photobleached to a single molecule above background, sifted using the signal-to-noise threshold. Data for individual particles are shown above the corresponding distribution in a jitter and box plot (showing median, interquartile range and min–max range).

 © The Author(s)

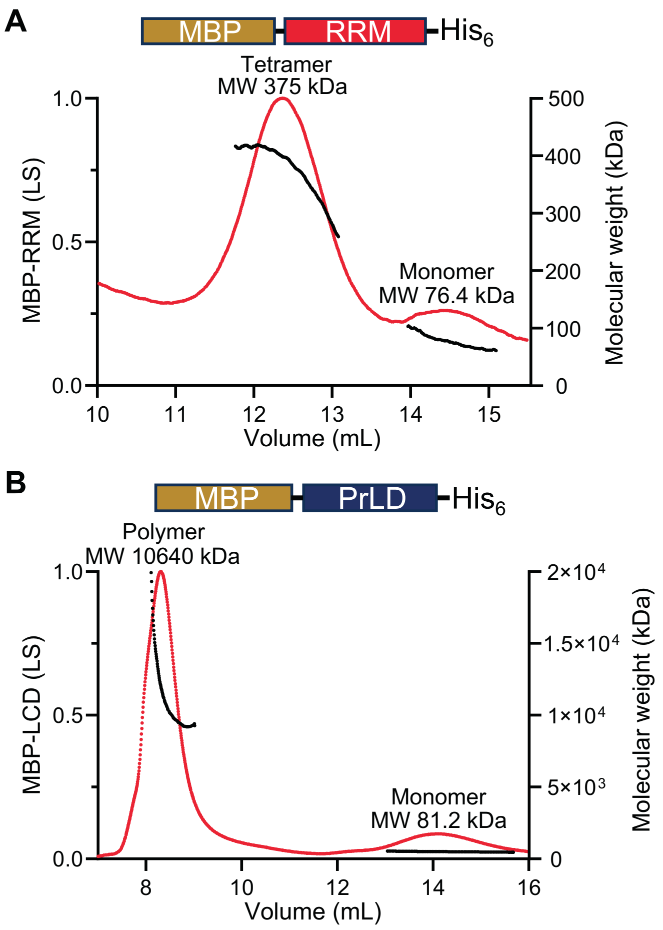

Figure EV3. SEC-MALS of purified FCA truncated proteins.

SEC-MALS of purified MBP-RRM (**A**) or MBP-PrLD (**B**). Line traces indicate molar mass as determined from MALS. The predicted molecular mass of MBP fused FCA truncated protein MBP-RRM and MBP-PrLD is approximately 72 kDa and 89 kDa, respectively.

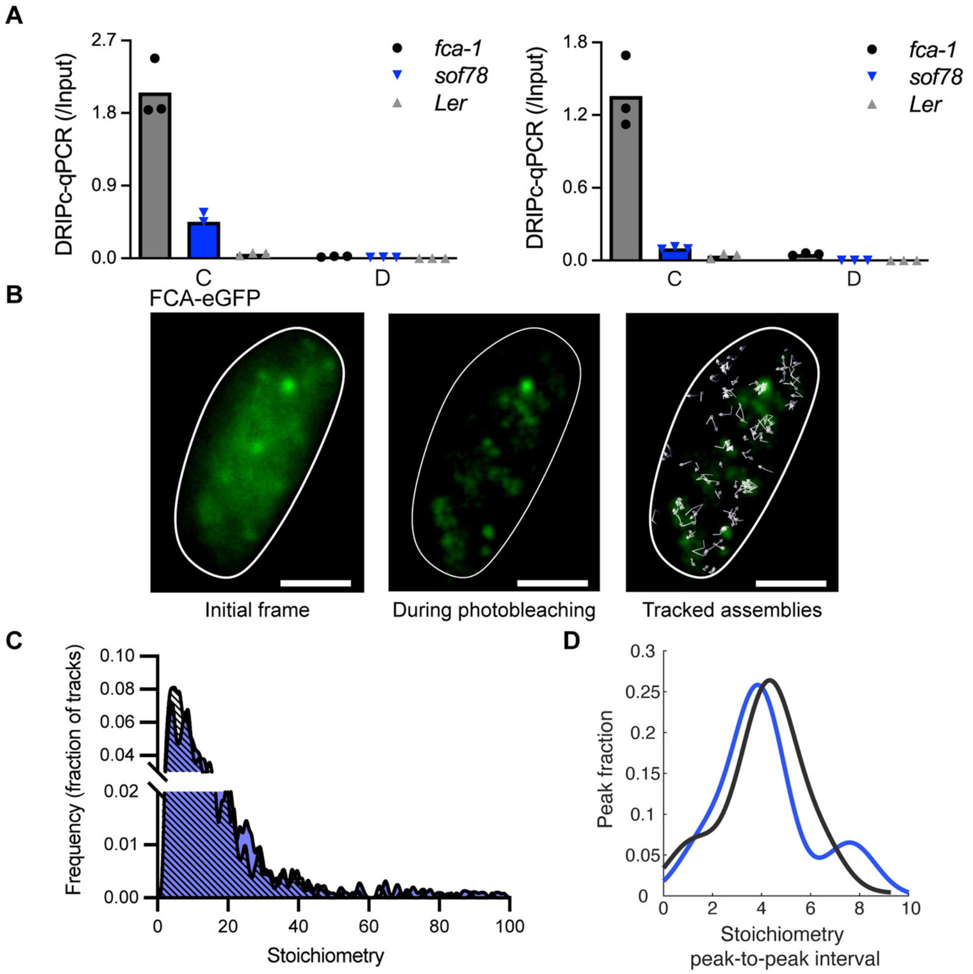

Figure EV4. The *COOLAIR* R-loop level and FCA-eGFP single-particle tracking in the *sof78* mutant.

(**A**) Two biological replicates of DRIPc–qPCR *COOLAIR* R-loop in the L*er*, *sof78* and *fca-1* genotypes. The *sof78* mutant carries a missense mutation in a region of FLL2 that is predicted to form a salt bridge, which influences FCA condensation (Fang et al, 2019). Data are means from three technical repeats. TSS, transcription start site. The primer sets used for DRIPc-qPCR (**C**, **D**) are the same as those shown in Fig. 4D. (**B**) An example of FCA-eGFP particle tracking at 10 ms/frame. The left panel shows the initial frame of the acquisition. The third panel features the same intermediate frame overlaid with tracks (white arrows) corresponding to the paths of each detected assembly throughout the acquisition. The nuclear boundary (white closed line) is obtained by blurring the initial frame and setting the Otsu intensity threshold. Scale bar: 2 µm. (**C**) Distributions of stoichiometry of individual FCA-eGFP (black) and FCA-eGFP/*sof78* (blue) particles. (**D**) Periodicity of FCA-eGFP (black) and FCA-eGFP/*sof78* (blue) particle stoichiometry. The stoichiometry in the figure is capped at 100. The single-particle tracking analysis is from three biologically independent experiments.

© The Author(s)

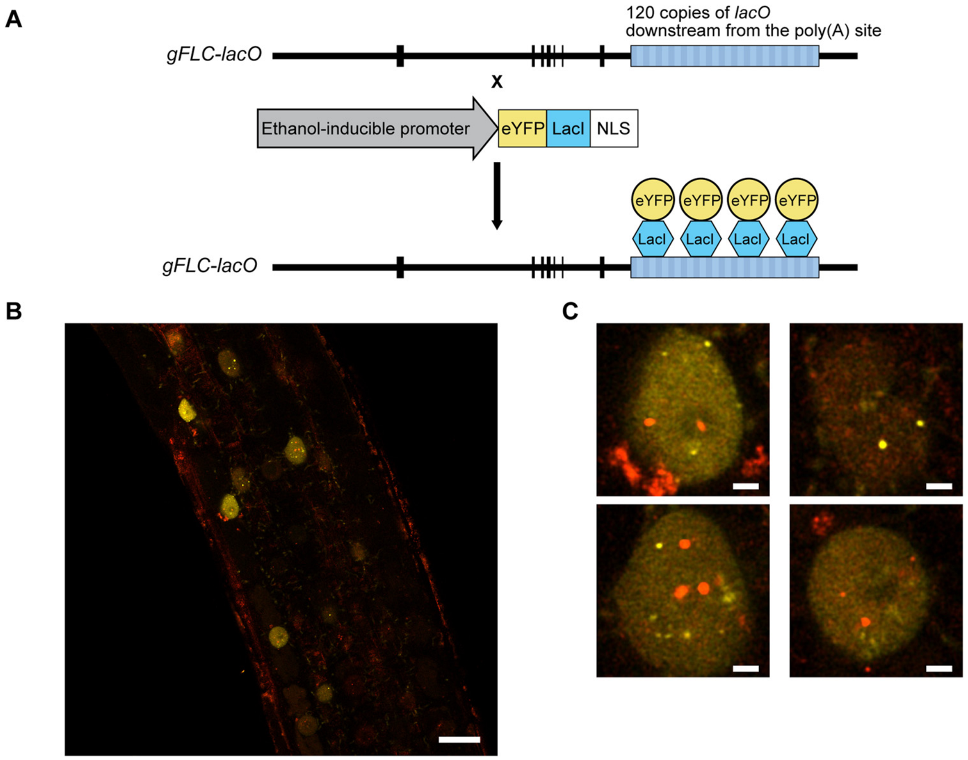

Figure EV5. Co-localization analysis of FCA condensates and *FLC-lacO*/eYFP-LacI.

(**A**) An illustration of the *FLC-lacO*/eYFP-LacI transgenic plants. (**B**) Single slice of Arabidopsis root Airyscan image showing expression of FCA-mScarlet-I (red) and *FLC-lacO*/eYFP-LacI (yellow) in nuclei. Scale bars: 20 µm. (**C**) Subsets of Airyscan images from (**B**) showing nuclei co-expressing FCA condensates (red) and *FLC-lacO*/eYFP-LacI (yellow). Scale bars: 2 µm. The FCA-mScarlet-I and LacI-eYFP signals were enhanced by shifting their dynamic ranges to 0–1099 and 0–4220 in EV5B, and to 0–1000 and 0–6000 in EV5C. The images were acquired on a Zeiss LSM980 using LD C-Apochromat 40x/NA 1.1 water-immersion objective. The fluorescent proteins, eYFP and mScarlet-I, were excited at 488 and 561 nm, respectively.
